# Rapid evaluation of COVID-19 vaccine effectiveness against symptomatic infection with SARS-CoV-2 variants by analysis of genetic distance

**DOI:** 10.1038/s41591-022-01877-1

**Published:** 2022-06-16

**Authors:** Lirong Cao, Jingzhi Lou, See Yeung Chan, Hong Zheng, Caiqi Liu, Shi Zhao, Qi Li, Chris Ka Pun Mok, Renee Wan Yi Chan, Marc Ka Chun Chong, William Ka Kei Wu, Zigui Chen, Eliza Lai Yi Wong, Paul Kay Sheung Chan, Benny Chung Ying Zee, Eng Kiong Yeoh, Maggie Haitian Wang

**Affiliations:** 1 JC School of Public Health and Primary Care, The Chinese University of Hong Kong, Hong Kong SAR, China; 2 CUHK Shenzhen Research Institute, Shenzhen, China; 3 Beth Bioinformatics Co. Ltd., Hong Kong SAR, China; 4 Li Ka Shing Institute of Health Sciences, The Chinese University of Hong Kong, Hong Kong SAR, China; 5 Department of Paediatrics, The Chinese University of Hong Kong, Hong Kong SAR, China; 6 Hong Kong Hub of Paediatric Excellence, The Chinese University of Hong Kong, Hong Kong SAR, China; 7 Department of Anaesthesia and Intensive Care and Peter Hung Pain Research Institute, The Chinese University of Hong Kong, Hong Kong SAR, China; 8 State Key Laboratory of Digestive Disease, The Chinese University of Hong Kong, Hong Kong SAR, China; 9 Department of Microbiology, The Chinese University of Hong Kong, Hong Kong SAR, China; 10 Centre for Health Systems and Policy Research, The Chinese University of Hong Kong, Hong Kong SAR, China; 11 Stanley Ho Centre for Emerging Infectious Diseases, The Chinese University of Hong Kong, Hong Kong SAR, China

**Keywords:** Infectious diseases, Computational models

## Abstract

Timely evaluation of the protective effects of Coronavirus Disease 2019 (COVID-19) vaccines against severe acute respiratory syndrome coronavirus 2 (SARS-CoV-2) variants of concern is urgently needed to inform pandemic control planning. Based on 78 vaccine efficacy or effectiveness (VE) data from 49 studies and 1,984,241 SARS-CoV-2 sequences collected from 31 regions, we analyzed the relationship between genetic distance (GD) of circulating viruses against the vaccine strain and VE against symptomatic infection. We found that the GD of the receptor-binding domain of the SARS-CoV-2 spike protein is highly predictive of vaccine protection and accounted for 86.3% (*P* = 0.038) of the VE change in a vaccine platform-based mixed-effects model and 87.9% (*P* = 0.006) in a manufacturer-based model. We applied the VE-GD model to predict protection mediated by existing vaccines against new genetic variants and validated the results by published real-world and clinical trial data, finding high concordance of predicted VE with observed VE. We estimated the VE against the Delta variant to be 82.8% (95% prediction interval: 68.7–96.0) using the mRNA vaccine platform, closely matching the reported VE of 83.0% from an observational study. Among the four sublineages of Omicron, the predicted VE varied between 11.9% and 33.3%, with the highest VE predicted against BA.1 and the lowest against BA.2, using the mRNA vaccine platform. The VE-GD framework enables predictions of vaccine protection in real time and offers a rapid evaluation method against novel variants that may inform vaccine deployment and public health responses.

## Abstract (web summary)

A model that predicts the effectiveness of COVID-19 vaccines against circulating SARS-CoV-2 variants, before the acquisition of real-world effectiveness data, may help guide more rapid public health and research responses to new variants of concern.

## Main

Vaccination is a crucial measure to control the scale of SARS-CoV-2 transmission and mitigate the severity of COVID-19. To date, 38 vaccines against SARS-CoV-2 are in early use or have been approved for application in the general population^1^. However, the protective effect of the various vaccine products is challenged by new genetic variants. VE against COVID-19, which measures the relative reduction of risk for a disease outcome in clinical trials or in the general population, exhibited a wide range of variation, from −2.7% to 97.2%^2,3^.

Several factors may contribute to the variations in VE that make it difficult to directly interpret the protective effect of vaccines. The notable contributors include the technology platforms, calendar period of studies, the target population, dosing interval, differences in study protocols and background risk of COVID-19, among others. The various vaccine technology strategies generated non-identical immune responses to provide protection against SARS-CoV-2 infection^4^. For instance, the LNP-mRNA vaccine, mRNA-1273, induces spike (S)-specific IgG, high T_H_1 cell responses, low T_H_2 cell responses and CD8^+^ T cell responses^5,6^, whereas the inactivated virus vaccine, CoronaVac, elicits robust CD4^+^ and CD8^+^ T cell responses to the structural proteins, including S, nucleocapsid (N), envelope (E) and matrix (M), in addition to humoral responses^7,8^. Among all the influencing factors, emerging genetic variants relative to the vaccine strain play a critical role in determining vaccine effectiveness. Serology studies showed that neutralizing activity against the Omicron variant decreased substantially in recipients of two COVID-19 vaccine doses^9,10^. Viral structure studies demonstrated that the amino acid substitutions in the receptor-binding domain (RBD) and N-terminal domain (NTD) alter virus–host cell interactions and reshape antigenic surfaces of the major neutralizing sites, leading to immune evasion^9,11–14^. Although the mechanisms of immune escape caused by the new mutations are being elucidated in experimental studies, an integrative framework to quantify the effect of genetic mismatch on VE would be instrumental for efficient evaluation of vaccine protection for any country in real time.

In this study, we evaluated the link between genetic mismatch of circulating SARS-CoV-2 viruses and reported COVID-19 VE from population studies. Based on our bioinformatics approach previously established for influenza viruses^15,16^, we tailored the VE estimation framework for COVID-19 by controlling the clustered random variation of technology platforms or manufacturers using a mixed-effects model. Through extensive analysis of publicly reported VE studies and genetic sequences, we showed that a substantial proportion of the change in VE could be explained by GD, and we proposed an efficient approach to evaluate vaccine protection against symptomatic COVID-19.

## Results

GD, or genetic mismatch, is calculated by the average Hamming distance on the RBD of the genome of the circulating viruses to the vaccine strain during the timeframe of VE studies. VE data used are detailed in Supplementary Table 1. The prediction method for VE was constructed through a mixed-effects model using GD as the main predictor, controlling for the confounding variables, including the midpoint (days) since the second dose and age group of the study. Particularly, variations in VE caused by technology platform or manufacturer were controlled by random effect in the mixed model (see Methods for details). In the following, we will first describe the variations in VE and GD by vaccine platform and then investigate their relationship.

### VE and GD distributions by vaccine platform

**Fig. 1:**
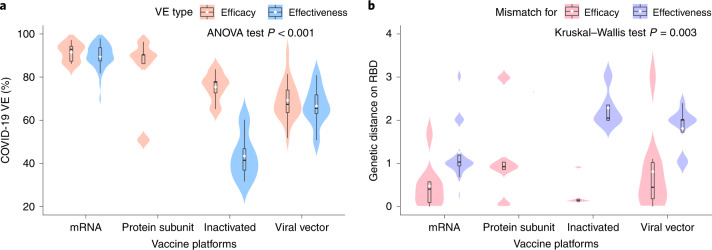
Comparison of COVID-19 VE and genetic mismatch across vaccine platforms. **a**, Distribution of the VE estimates for different platforms. The VE of mRNA and protein subunit vaccines are higher than other vaccines (two-sided ANOVA test *P* = 2.2 × 10^−14^, *n* = 78). **b**, Distribution of genetic mismatch on RBD for different vaccine technologies. Genetic mismatch is the lowest for mRNA vaccines (two-sided Kruskal–Walls test *P* = 0.003, *n* = 78). In the box plots, the middle bar indicates the median; the white dot indicates the mean; and the boundaries are Q1 and Q3. Whiskers of the box plot are extended to Q3 + 1.5× interquartile range (IQR) and Q1 − 1.5× IQR.

**Extended Data Fig. 1:**
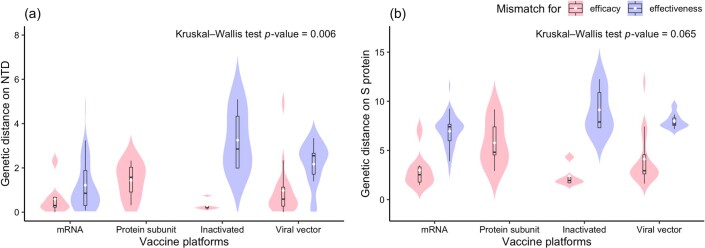
Distribution of genetic mismatch on the NTD and complete S protein. The genetic mismatch on the NTD and S protein was measured. The results show that the genetic mismatch is lowest for mRNA vaccines (Kruskal-Wallis test: two-sided *P* of NTD = 0.006, two-sided *P* of S protein = 0.065; n = 78). In the box plots, the middle bar indicates the median, the white dot indicates the mean, and the boundaries are Q1 and Q3. Whiskers of the box plot are extended to Q3 + 1.5×IQR and Q1 − 1.5×IQR.

VE and GD of the four vaccine platforms with authorized use are compared in Fig. 1. Within each vaccine platform, the vaccine effectiveness is generally lower compared to the efficacy outcome (Fig. 1a), whereas, in terms of genetic mismatch (Fig. 1b and Extended Data Fig. 1), the vaccine effectiveness cohort encompasses larger genetic mismatch relative to the vaccine efficacy cohorts. The result indicates that genetic mismatch had increased during the mass vaccination phase compared to the earlier clinical trial periods. This could be due to the accumulation of virus mutations through time, as well as the generally longer evaluation period of the effectiveness studies compared to the efficacy trials. Across the technology platforms, vaccine protection (efficacy/effectiveness) shows considerable difference (ANOVA test *P* < 0.001; Fig. 1a). The mRNA vaccines reported the highest mean VE of 90.0% (95% confidence interval (CI): 88.2–91.8, *n* = 39), followed by the protein subunit vaccine (82.9%) (95% CI: 67.0–98.8, *n* = 5), viral vector vaccines (68.5%) (95% CI: 64.8–72.1, *n* = 24) and inactivated vaccine (59.6%) (95% CI: 47.8–71.3, *n* = 10). Interestingly, the genetic mismatch of these platforms shows a perfect reverse trend, of which the mRNA vaccines cohorts correspond to the smallest mismatch, and the other platforms exhibit larger mismatches. This might also be contributed by the timeframe of the vaccine evaluations for these platforms, in which the mRNA trials were the earliest to complete and corresponded to a more homogeneous viral population. The genetic mismatch summarizes the deviation of genetic variants with respect to the vaccine strains, accounting for time, locations and multiple strain co-circulation, for vaccine evaluation at population level using sequencing data.

### Relationship between vaccine protection and GD

**Fig. 2:**
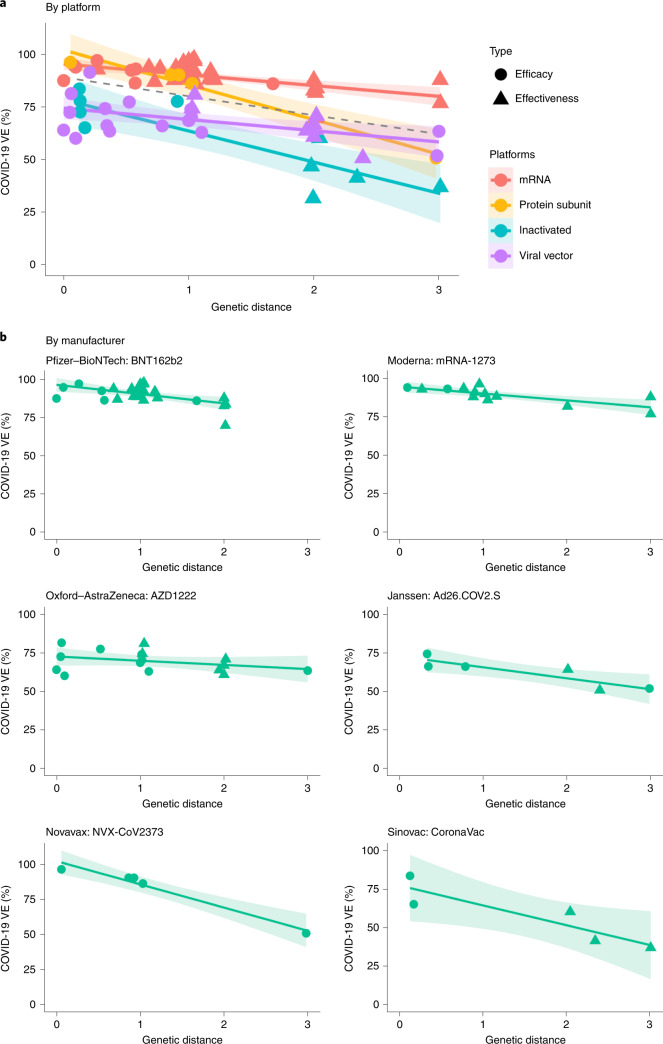
The relationship between VE and GD of the circulating SARS-CoV-2 strains to the vaccine strain on RBD. **a**, Negative linear relationships between VE and GD for different vaccine platforms (*P* = 0.038, R^2^ = 86.3%). The dashed line was fitted by all data points. **b**, Negative linear relationship between VE and GD for each vaccine product (*P* = 0.006, R^2^ = 87.9%). The two-sided *P* value was obtained from the mixed-effects model. The colored lines were fitted by data points of each platform. The shaded area indicates 95% CI.

**Extended Data Fig. 2:**
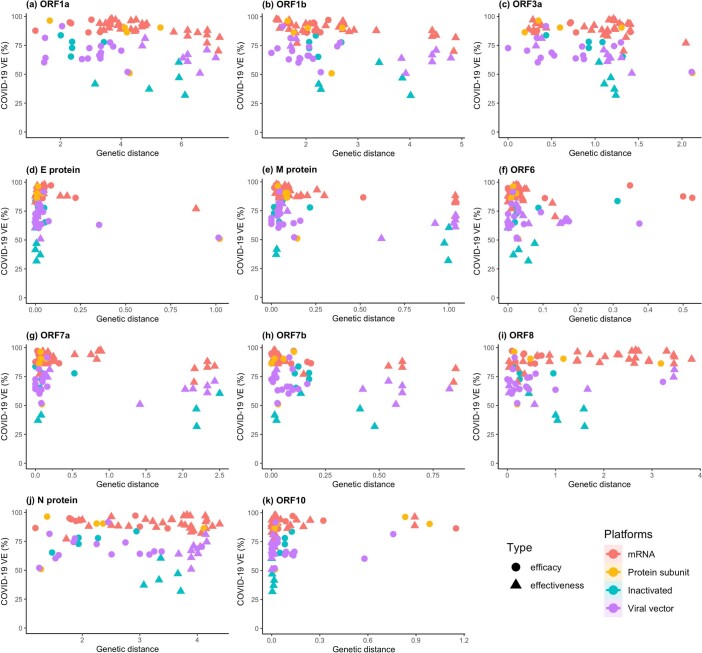
Scatterplot of the observed VE and genetic distance on the non-S proteins of SARS-CoV-2. The same analysis under the null hypothesis to explore the association of VE with genetic distance was performed on the structural proteins containing envelope (E), membrane (M) and nucleocapsid (N); ORF1ab; accessory proteins containing ORF3a, ORF6, ORF7a, ORF7b, ORF8 and ORF10 proteins. No significant relationship with VE was observed.

**Extended Data Fig. 3:**
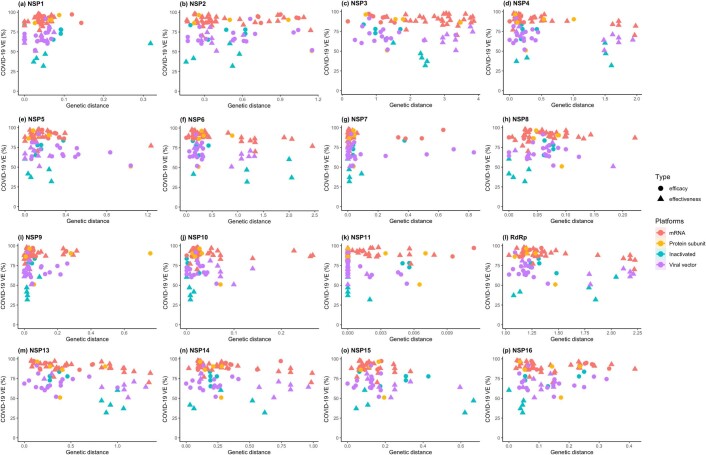
Scatterplot of the observed VE and genetic distance on the non-structural proteins (NSPs). The ORF1ab polyprotein is composed of 16 non-structural proteins (NSPs). The genetic distance of each NSP was also calculated and no relationship with VE was observed.

**Extended Data Fig. 4:**
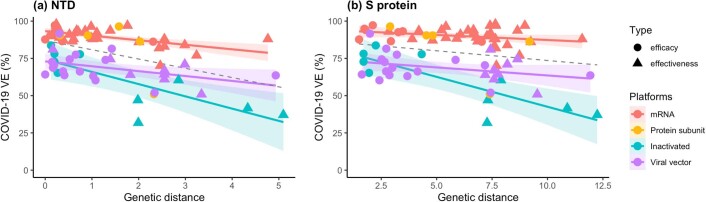
The relationship between VE and genetic distance on the NTD and S protein. Panels (a-b): negative linear relationships between VE and genetic mismatch for NTD (*P* = 0.086, *R*^2^ = 75.8%), and full-length sequence (*P* = 0.082, *R*^2^= 78.4%), respectively. The two-sided *P* was obtained from the mixed-effects model. The colored lines were fitted by data points of each platform. The shaded area indicates 95% CI.

Next, we explored the effect of GD on vaccine protection. At most, 86.3% of the variations in VE can be explained by the GD measure, controlling for the random effects of vaccine technology platforms (Fig. 2a and Supplementary Table 2), and 87.9% of the variation can be explained (Fig. 2b and Supplementary Table 3) when the random effects of six major vaccine products (BNT162b2, mRNA-1273, AZD1222, Ad26.COV2.S, NVX-CoV2373 and CoronaVac) were controlled. Among the candidate genomic regions, genetic mismatch on the RBD demonstrates the strongest influence on vaccine protection, whereas the GD of the non-S proteins shows no association with VE (Extended Data Figs. 2 and 3). For every residue substitution on the RBD, the VE would reduce by an average of 5.2% (95% CI: 2.4–8.0) for mRNA vaccines, 6.8% (95% CI: 4.2–9.4) for viral vector vaccines, 14.3% (95% CI: 9.4–19.2) for protein subunit vaccines and 15.8% (95% CI: 12.4–19.3) for inactivated vaccines (*P* = 0.038) (Supplementary Table 2). The NTD and S protein demonstrate weaker per-amino-acid substitution association with VE (*P* = 0.086 and *P* = 0.082, respectively) (Extended Data Fig. 4 and Supplementary Table 4). When no genetic mismatch is present, VE for the mRNA vaccines is expected to be 95.8% (95% CI: 92.0–99.5), estimated by the RBD region; the protein subunit vaccine’s expected VE is similar; and the inactivated and viral vector vaccines are expected to exhibit a systematically lower VE by 17.3% and 20.6% compared to the mRNA vaccines. The estimates using the manufacturer-based model can be found in Supplementary Table 3.

### VE-GD model assessment by validation data

**Fig. 3:**
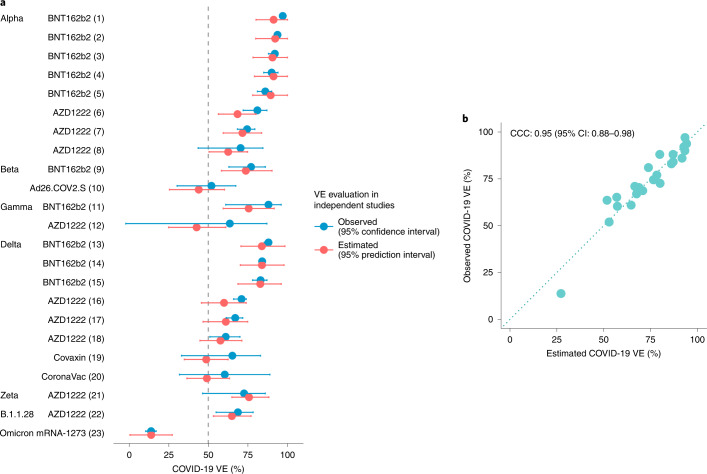
Prediction of VE based on GD. The VE-GD model was trained using all non-variant-specific VEs, and estimations were made on all (*n* = 23) variant-specific VEs. **a**, Estimated and observed VE for variants by vaccine product. Source of the VE data is indexed behind vaccine product names, available in Supplementary Table 5. **b**, Calibration plot for the prediction outcome in validation data. The predicted VEs are close to the observed VEs with a CCC of 0.95 (95% CI: 0.88–0.98).

The VE-GD relationship can be used to make predictions on VE by vaccine type. A total of 57 VE data were used for model training and 23 variant-specific VE data for validation (Supplementary Table 5). In Fig. 3a, the predicted and observed VEs for the genetic variants are overlayed. The calibration plot (Fig. 3b) shows a close matching, and the concordance correlation coefficient (CCC) reaches a high level of 0.95 (95% CI: 0.88–0.98). Against the Delta variant (B.1.617.2), the estimated VE is 82.8% (95% prediction interval: 68.7–96.0) and 61.1% (95% prediction interval: 46.7–74.9) by the mRNA and viral vector vaccines, respectively (Fig. 3a). These estimates are supported by the observed VE against the Delta variant: the mRNA vaccine BNT162b2 and the viral vector vaccine AZD1222 provided 83% (95% CI: 78–87) and 67.0% (95% CI: 61.3–71.8) protection, respectively^17,18^. The predicted VE is 89.4% (95% prediction interval: 78.1–100.0) for the Alpha variant and 73.7% (95% prediction interval: 58.3–90.2) for the Beta and Gamma variants by the BNT162b2 and mRNA-1273 vaccines, close to the observed VE of 86% (95% CI: 81–90) and 77% (95% CI: 63–86), respectively^19^. Against the Omicron variant, the model predicted an expected VE of 14.0% (95% prediction interval: 0.7–27.3) in California in late 2021, and the observed value was 13.9% (95% CI: 10.5–17.1) for the mRNA-1273 vaccine^20^. These validation results demonstrate high predictive feasibility of using genetic mismatch to estimate vaccine performance.

### Prediction for variants and Omicron sublineages without known VEs

**Fig. 4:**
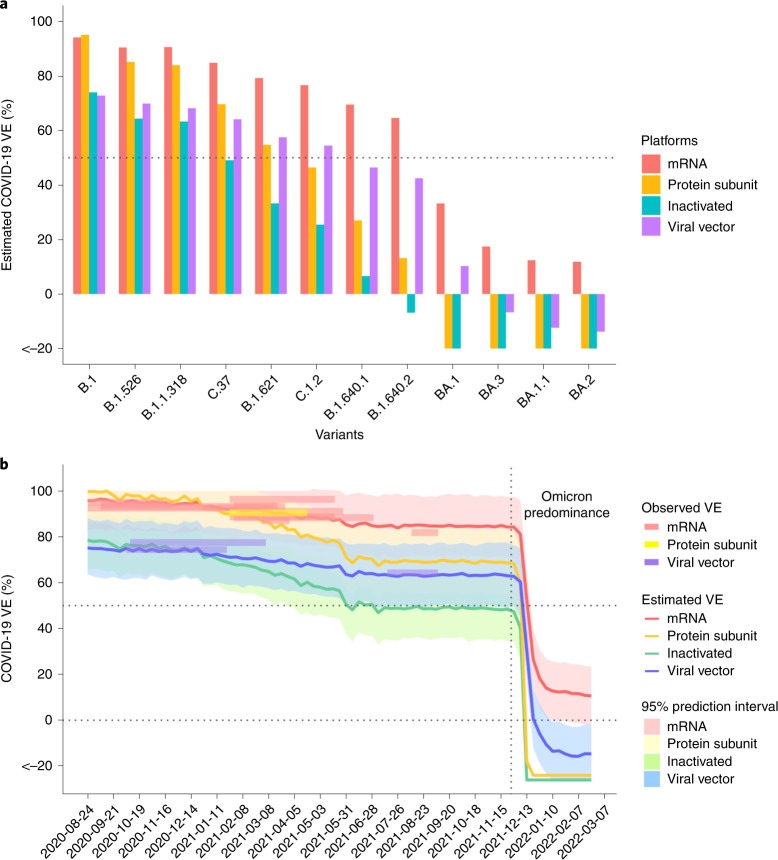
Prediction for SARS-CoV-2 genetic variants with unknown VEs including the Omicron sublineages and in serial cross-sectional sequencing data. **a**, Predicted VEs for specific variants/sublineages without observed VEs. Omicron sublineages: BA.1, BA.1.1, BA.2 and BA.3. **b**, VEs in California were estimated at weekly intervals for different vaccine platforms. The surveyed VEs from clinical trials or observational studies during the same period are overlaid on the trend curve as colored rectangles for reference. The declining trend of estimated VE captures the influence of virus evolution on population-level immune protection. During the Omicron predominance period, a cliff-like drop of VE is depicted. The shaded areas are 95% prediction interval. The vertical dashed line marks the date of 26 November 2021, which is the earliest time of the Omicron appearance in these data. The top horizontal dashed line marks the 50% efficacy threshold.

Next, we fitted the model with all available data and predicted VE against circulating variants as well as the Omicron sublineages for which there are no observed VE data at the time of writing (Fig. 4a). Interestingly, among the four sublineages of Omicron (BA.1, BA.1.1, BA.2 and BA.3), the expected VEs vary between 11.9% for BA.1 and 33.3% for BA.2, using the mRNA vaccines. This might contribute to the considerable variations in VEs for Omicron reported from observational studies, whose cohorts might have been infected by divergent Omicron sublineages, in addition to differences in immune history. The model predicts that VEs against variants of concern (VOCs) or variants of interest (VOIs) other than the Omicron, such as the Lambda and Mu variants, are expected to be above 50% within 3 months after the second dose of an mRNA vaccine; however, the VEs of inactivated vaccines against symptomatic infection are predicted to wane most under the challenge of new genetic variants.

### Depicting trend of VE in serial cross-sectional sequencing data

We demonstrated the application of predicting VE in real time against the circulating virus in a given geographical region, using California as an example. Sequencing data of virus isolates from California were downloaded from public databases. VEs were estimated for the major vaccine platforms at weekly intervals by GD in the serial cross-sectional sequencing data (Fig. 4b). In general, a decreasing trend of VE is depicted, with a sharp drop after the Omicron predominance since December 2021. The observed VEs from clinical trials and observational studies conducted during the period in the United States are overlaid on the prediction outcomes for reference^2,21–32^.

### Exploration of candidate vaccine strains

**Fig. 5:**
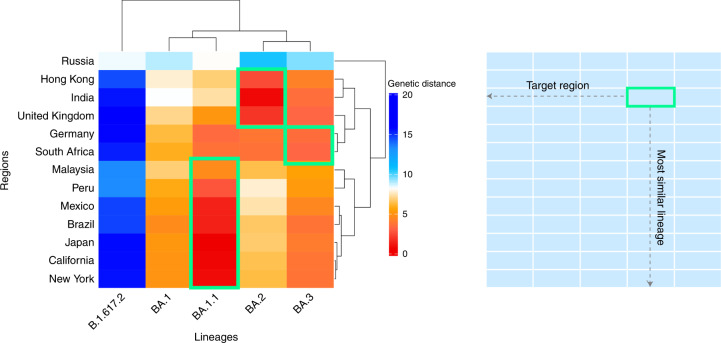
Clustering of regions by GD between circulating strains and candidate vaccine strains during January and February 2022. The candidate strain that gives a lowest genetic mismatch for geographical regions is highlighted in the green box. Rows: target geographical regions; columns: candidate vaccine strain (B.1.617.2: Delta; BA.1–BA.3: Omicron sublineages). The figure shows that the Omicron sublineages can match to the epidemic viruses in most regions, but the dominant sublineages are not the same.

**Extended Data Fig. 5:**
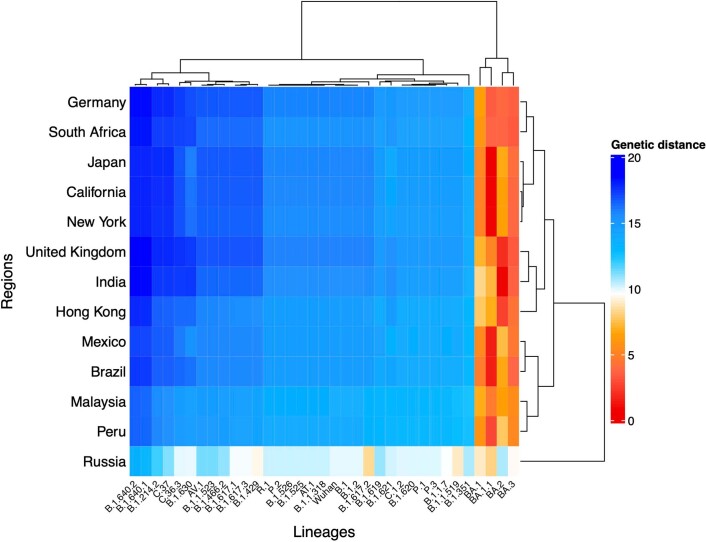
Clustering of regions by circulating strains similarities to SARS-CoV-2 genetic variants. Genetic mismatch of genetic variants to the local circulating virus during January and February 2022. The best candidate vaccine antigen for a geographical region measured by genetic distance is shown by dark red. Rows: target geographical regions; columns: candidate vaccine strains. The figure shows that the Omicron sublineages can match to the epidemic viruses in most regions, but the dominant sublineages are not the same.

We further explored the possibility of developing region-specific vaccines and how well they would match the circulating virus profiles. We investigated the optimal candidate vaccine strains for 13 regions, including the United Kingdom, Germany, South Africa, Russia, India, Hong Kong, Malaysia, Japan, California, New York, Mexico, Peru and Brazil. Based on the GD between the vaccine strain and observed viruses circulating in a given region and period, hierarchical clustering of regions was performed to show the similarity of vaccine mismatches (Fig. 5 and Extended Data Fig. 5). We found that, although the Omicron sublineages can match to epidemic viruses in all investigated regions except for Russia during January and February 2022, the dominant sublineages were not the same in these regions. This suggests that updating vaccine compositions with a single genetic variant might not be sufficient for matching the distribution of global viral population.

## Discussion

As novel variants of SARS-CoV-2 keep emerging in the ongoing pandemic, rapid assessment of vaccine performance in populations is crucial to inform public health and clinical responses. This study established an efficient computational framework to estimate COVID-19 VE against symptomatic infection using viral sequence data. We show that the predicted VEs against genetic variants are close to the observed outcomes. The framework has several advantages. First, it enables prediction of VE against novel variants using existing virus surveillance networks to derive a rapid estimate; thus, it could inform timely public health preparedness. Second, it provides an integrated measure to facilitate the interpretation of vaccine effects, which accounts for the potential confounding effects of time and location related to genetic evolution. Third, through mixed-effects modeling, the framework controls for variations by vaccine type, providing a consistent and adaptable prediction framework for inclusion of multiple vaccine platforms and manufacturers.

Among candidate genomic regions, the RBD region exhibits the strongest statistical association with VE. Weaker associations between VE and GD were detected for NTD and the entire S protein. These findings are also supported by biological evidence. The RBD is the major target for neutralizing antibodies that interfere with viral receptor binding^33,34^. The NTD is reported to be the target of 5–20% of S-specific monoclonal antibodies from memory B cells against SARS-CoV-2 (refs. ^35,36^).

Recent studies have investigated the use of neutralization titer as a predictor of vaccine efficacy^37–39^; however, the neutralizing results against SARS-CoV-2 genetic variants showed varying outcomes. The vaccine protection against the B.1.351 variant reduced from 95.0%^2^ to 75.0%^40^ for BNT162b2 in early 2021. Due to differences in standardization and cohorts, one neutralization study showed that the titer against B.1.351 is 7.6-fold and nine-fold lower compared to the early Wuhan-related Victoria variant in the BNT162b2 vaccine serum and AZD1222 vaccine serum, respectively^41^, whereas another experiment reported a 2.7-fold decrease in neutralization titers against the B.1.351 lineage in the BNT162b2-elicited serum^42^. Similar results have also been observed for the Omicron variant^43–45^. The varying neutralization results increase the challenge of inferring vaccine performance solely by neutralization levels. The association of neutralization with protection across studies showed that neutralizing antibodies might not be deterministic in mediating protection, and the effect of other vaccine-induced immune responses also need to be quantified. This work uses an alternative angle to bridge the link between genetic variations and population-level vaccine responses. Further investigations are needed to integrate potential correlates of vaccine protection and improve the existing framework.

Although 42% of the world population has not completed the full vaccine primary series up to this date^46^, additional booster doses of vaccine are being rolled out in many places. Neutralization activity after the booster can be restored to a higher level for a short period of time. BNT162b2 immune sera of individuals who received only two doses had a low ability to neutralize the Omicron variant, whereas a third dose of the BNT162b2 increased the Omicron-neutralizing titer 23-fold relative to their level at 21 days after the second dose^47^. Similar results have been reported for the mRNA-1273 vaccine^48^. The booster-enhanced neutralizing level against Omicron was lower than that against the Beta, Delta and Wuhan strains and declined faster than those against the D614G variant^47,48^. Recent studies showed that the VE against symptomatic infection of Omicron is restored up to near 50% after the booster. In Qatar, the VE against symptomatic Omicron infection was 56.6% and 53.1% for the BNT162b2 and mRNA1732 vaccines, respectively, 1 month after the third dose^49^; and, in Israel, the VE against symptomatic Omicron infection was 43% and 31% for BNT162b2 and mRNA1273, respectively, 1 month after the fourth dose among healthcare workers^50^. The flexible VE-GD framework proposed here could be further extended to account for the booster’s protection as more effectiveness data of homologous and heterologous booster studies are available.

VE against infection is generally lower compared to the VE against symptomatic infection. For instance, in the Coronavirus Efficacy (COVE) phase 3 trial of the mRNA-1732 vaccine, the VEs for infection and symptomatic infection are 82.0% and 93.2%, respectively^51^. In view of waning immunity, a systematic review including 78 VE studies up to 2 December 2021 showed that the VE dropped by 21.0% (95% CI: 13.9–29.8) and 24.9% (95% CI: 13.4–41.6) against infection and symptomatic infection, respectively, 6 months after the second dose, aggregating the data from several vaccine platforms^52^. VE against severe disease or hospitalization showed longer preservation compared to the protection against symptomatic infection. In Qatar and Canada, the VE against hospitalization due to infection with the Alpha, Beta and Delta variants among all age groups was above 90% after the second dose of the mRNA-1273, BNT162b2 and AZD1222 vaccines^53–56^. VE against hospitalization with Delta infection remained at above 80% in the United Kingdom 20 weeks after vaccination with the BNT162b2 and AZD1222 vaccines^57^. In Qatar and South Africa, VE against hospitalization was in the range of 70–80% during the Omicron predominance within 6 months after the second dose for mRNA vaccines^49,58^.

Previously, the effect of genetic diversity on vaccine efficacy was investigated by sieve analysis, originating in the study of the human immunodeficiency virus 1 (HIV-1) vaccines^59–61^. Sieve analysis compares the infection strains between vaccinated and unvaccinated individuals and estimates the odds ratio of a viral strain type to penetrate the vaccine protection barrier. The sieve method requires individual-level data of virus isolate sequences and infection outcome of trial participants, whereas the model proposed in this study uses viral sequences in the general population and integrates multiple VE studies. Other studies have considered the proportion of genetic mismatch in the dominant epitope region to account for variations in the VE against influenza viruses^62,63^, whereas the VE-GD model in this report provides a unified framework to account for multiple genes and vaccine types.

This study has several limitations. The scope of inference is subject to the range of VE studies included in model fitting; thus, the VE estimated is presumably for a time close to the second vaccine dose. In model estimation, the effect of waning immunity on VE was controlled by a proxy time variable at population level, and the VE decline corresponding to time was estimated to be 2.4% (95% CI: 1.0–3.8) per 30 days for mRNA vaccines. This estimation is in line with the phase 2/3 efficacy trial of the BNT162b2 vaccine through 6 months of follow-up^32^, which showed an average decline of 2.5% per month by comparing the VE after 4–6 months to VE within 2 months since the second dose. The exact relationship between time and waning of host immunity will be calibrated in individual-level data, in which the main variable of interest is time-to-infection. For these analyses, including the genetic mismatch information would be helpful to control for the genetic variant’s effect on vaccine breakthrough alongside waning of host immunity. Second, VE prediction in this study only considered the GD of vaccine strain to circulation strains, and the effect of prior infection on vaccine protection was not captured. Studies showed that natural infection, either before or after vaccination, substantially increased vaccine protection for symptomatic infection and hospitalization during the Beta-predominant and Delta-predominant periods^64^ and against the Omicron variant by the mRNA vaccine^65,66^. As more hybrid immunity data become available, the mixed-effects prediction model could be extended to account for this additional level of variation. Moreover, bias might occur if sequences in databases disproportionately represented regions with known circulation of a given variant. Enhanced efforts are needed to ensure better geographical representativeness of available SARS-CoV-2 sequences. Despite these limitations, we demonstrated a robust relationship between genetic mismatch and VE, which we validated using independent data.

To conclude, this work developed a modeling framework integrating data from genetics and epidemiological studies for estimating COVID-19 vaccine effectiveness against a specific variant or for a particular cohort in a given period and region. Rapid assessment of VE against an evolving pathogen can be a useful instrument to inform vaccine development, distribution and public health responses.

## Methods

### VE data

VE is calculated by (1 − RR) × 100), where RR is the relative risk of a disease outcome in the vaccinated group compared to the unvaccinated group. Vaccine efficacy is measured in randomized controlled trials, whereas vaccine effectiveness is obtained from observational studies. VE reports before 24 December 2021 were collected from published articles and preprint articles. Inclusion criteria for the vaccine effectiveness studies include: target population is a cohort without special conditions; the primary outcome is symptomatic COVID-19 infection after the second vaccine dose; and the study period of VE evaluation is clearly reported. A total of 78 VE data from 49 studies were obtained for estimating the effect size of GD, among which were 33 efficacy data and 45 were effectiveness data. The vaccine efficacy studies include 28 phase 3 trials, one phase 2 trial and four phase 2/3 trials. The vaccine effectiveness studies include 16 cohorts and 29 case–control studies. Detailed information of VE studies is available in Supplementary Table 1.

### Genetic sequences

Human SARS-CoV-2 strains with collection dates ranging from 4 August 2020 to 6 March 2022 were retrieved from the Global Initiative on Sharing All Influenza Data (GISAID) EpiCoV database^67^. All available sequences that matched to the period and locations of the clinical trials or observational studies totaled 1,984,241 full-length genome sequences from 31 geographical regions. The sources of SARS-CoV-2 sequences involved in this study are reported in the Supplementary Acknowledgement Table. Strains with duplicated names and unclear collection time of samples were removed. Multiple sequence alignment was performed using MAFFT (version 7). The ‘Wuhan-Hu-1’ genome (GenBank NC_045512.2 or GISAID EPI_ISL_402125) was set as the reference sequence. The variants involved in this study are summarized in Supplementary Tables 6 and 7. Lineage classification for sequences was referenced from the GISAID.

### Statistical methods

#### GD

Following our previous framework developed for influenza virus^15^, let $X = \{ x_{ij} \}$ denote the *i*-th sample from the GISAID database collected for a target population, where *i* = 1,…, *n*, *j* = 1,…, *J*; and let $V = \{ v_j \}$ denote the vaccine strain applied in the target population, where index *j* indicates the *j*-th codon position in the sequence. Denote the amino acids in a given genomic region as $W = \left\{ {w_k^{}} \right\}$, where *k* is the index for codon positions contained in the segment, *k* = 1, …, *K*, 0 ≤ *K* ≤ *J*. Suppose the Hamming distance is used as a basic measure of dissimilarity between two sequences, the vaccine genetic distance (*d*) calculated for the target population is:

1 $$d = \mathop {\sum }\limits_{i = 1}^n d_i/n = \mathop {\sum }\limits_{i = 1}^{n_{}} \mathop {\sum }\limits_{k = 1}^K I\left( {v_{w_k} \ne x_{w_k}} \right)/n.$$

Thus, the *d* summarized the average amino acids mismatch of circulating strains versus the vaccine strain based on a given genomic segment in a target population. In this study, we considered a wide range of candidate *W*, including the RBD, NTD and S, E, M, N, ORF1ab and accessory proteins. A schematic representation of the SARS-CoV-2 genome and the structure of S protein are available in Supplementary Figs. 1 and 2. All vaccine strains are based on the Wuhan strain isolated in January 2020. When the target population is composed of individuals infected with multiple co-circulating variants, the *d* captures the average mismatch over all co-circulating variants in the cohort, whereas, when the target population is a single genetic variant, *d* captures the variant-specific distance.

#### The VE-GD mixed-effects model

A two-level mixed-effects model was adopted to account for the random effect associated with vaccine type (technology platform or manufacturer). The genetic distance, *d*_*ij*_, is the main predictor variable for study *i* and vaccine type *j*, *i* = 1,…, *n*_*j*_, and *n*_*j*_ is the number of studies for vaccine type *j*. Therefore, the following random intercept and random slope model is specified for the VE response *Y*_*j*_:

2 $$Y_j = X_j\beta + Z_ju_j + \varepsilon _j$$

In the equation, *X*_*j*_ is the covariate matrix of fixed factors, and *β* is the fixed effect vector. $Z_j = [1,\,{{{\boldsymbol{d}}}}_j]$ is the matrix containing a unit vector and the *n*_*j*_-length genetic distance vector ***d***_*j*_; and $u_j = (u_{0j},u_{1j})^T$ is composed of a random intercept variable $u_{0j}$ and a random slope variable $u_{1j}$. $u_j\sim N(0,D)$, where *D* is a variance component matrix. The fixed factors include the age category of the study, midpoint (days) after the second dose extracted from each study and the genetic distance ***d***_*j*_. $\varepsilon _j\sim N(0,R_j)$ is the error term of the mixed-effects model, $R_j = \sigma ^2I_{n_j}$. The model was fitted using the R package lmerTest^68^. The prediction interval of the mixed-effects model was calculated using the R package merTools^69^. All analyses were performed using R statistical software (version 4.0.3). Statistical significance was declared if *P* < 0.05.

Model assessment was performed in a training–validation setting. A total of 23 variant-specific VEs were extracted from the data as the validation set (Supplementary Table 5). The model was fitted using the remaining 57 VEs (non-variant specific), and predictions were made for the genetic variants. The agreement between the predicted and observed VEs is measured by the CCC^70^.

### Reporting summary

Further information on research design is available in the Nature Research Reporting Summary linked to this article.

## Online content

Any methods, additional references, Nature Research reporting summaries, source data, extended data, supplementary information, acknowledgements, peer review information; details of author contributions and competing interests; and statements of data and code availability are available at 10.1038/s41591-022-01877-1.

## Supplementary information

Supplementary Information Supplementary Tables 1–7 and Supplementary Figs. 1 and 2

Reporting Summary

## Data Availability

All data used in this study are publicly available. Detailed information of VE outcomes is available in the Supplementary Materials. Viral sequence data were downloaded from the GISAID at http://platform.gisaid.org/, and the accession numbers are provided in the online Supplementary Acknowledgment Table (https://github.com/VaccineEffectivenessPrediction/COVID19-Vaccine-Effectiveness).

## Code availability

All code is freely available at https://github.com/VaccineEffectivenessPrediction/COVID19-Vaccine-Effectiveness.

## References

[1] Basta, N. E. & Moodie, E. M. M., on behalf of the VIPER (Vaccines, Infectious disease Prevention, and Epidemiology Research) Group COVID-19 Vaccine Development and Approvals Tracker Team. COVID-19 Vaccine Tracker. https://covid19.trackvaccines.org/

[2] Polack FP (2020). Safety and efficacy of the BNT162b2 mRNA Covid-19 vaccine. N. Engl. J. Med..

[3] Andrews, N. et al. Covid-19 vaccine effectiveness against the Omicron (B.1.1.529) variant. *N. Engl. J. Med*. **386**, 1532–1546 (2022). 10.1056/NEJMoa2119451 PMC8908811 35249272

[4] Dai L, Gao GF (2021). Viral targets for vaccines against COVID-19. Nat. Rev. Immunol..

[5] Jackson LA (2020). An mRNA vaccine against SARS-CoV-2—preliminary report. N. Engl. J. Med..

[6] Anderson EJ (2020). Safety and immunogenicity of SARS-CoV-2 mRNA-1273 vaccine in older adults. N. Engl. J. Med..

[7] Mok, C. K. P. et al. Comparison of the immunogenicity of BNT162b2 and CoronaVac COVID-19 vaccines in Hong Kong. *Respirology* **27**, 301–310 (2022). 10.1111/resp.14191 PMC8934254 34820940

[8] Melo-Gonzalez F (2021). Recognition of variants of concern by antibodies and T cells induced by a SARS-CoV-2 inactivated vaccine. Front. Immunol..

[9] Dejnirattisai W (2022). SARS-CoV-2 Omicron-B.1.1.529 leads to widespread escape from neutralizing antibody responses. Cell.

[10] Cheng, S. M. S. et al. Neutralizing antibodies against the SARS-CoV-2 Omicron variant BA.1 following homologous and heterologous CoronaVac or BNT162b2 vaccination. *Nat. Med*. **28**, 486–489 (2022). 10.1038/s41591-022-01704-7 PMC8940714 35051989

[11] McCallum M (2022). Structural basis of SARS-CoV-2 Omicron immune evasion and receptor engagement. Science.

[12] Cui Z (2022). Structural and functional characterizations of infectivity and immune evasion of SARS-CoV-2 Omicron. Cell.

[13] Cai Y (2021). Structural basis for enhanced infectivity and immune evasion of SARS-CoV-2 variants. Science.

[14] Gobeil, S. M.-C. et al. Effect of natural mutations of SARS-CoV-2 on spike structure, conformation, and antigenicity. *Science* **373**, eabi6226 (2021). 10.1126/science.abi6226 PMC8611377 34168071

[15] Cao, L. et al. In silico prediction of influenza vaccine effectiveness by sequence analysis. *Vaccine* **39**, 1030–1034 (2021). 10.1016/j.vaccine.2021.01.006 33483214

[16] Cao, L. et al. Differential influence of age on the relationship between genetic mismatch and A(H1N1)pdm09 vaccine effectiveness. *Viruses* **13**, 619 (2021). 10.3390/v13040619 PMC8065480 33916601

[17] Sheikh A, McMenamin J, Taylor B, Robertson C (2021). SARS-CoV-2 Delta VOC in Scotland: demographics, risk of hospital admission, and vaccine effectiveness. Lancet.

[18] Lopez Bernal J (2021). Effectiveness of Covid-19 vaccines against the B.1.617.2 (Delta) variant. N. Engl. J. Med..

[19] Charmet T (2021). Impact of original, B.1.1.7, and B.1.351/P.1 SARS-CoV-2 lineages on vaccine effectiveness of two doses of COVID-19 mRNA vaccines: results from a nationwide case-control study in France. Lancet Reg. Health Eur..

[20] Tseng, H.F. et al. Effectiveness of mRNA-1273 against SARS-CoV-2 Omicron and Delta variants. *Nat. Med*. **28**, 1063–1071 (2022). 10.1038/s41591-022-01753-y PMC9117141 35189624

[21] Pilishvili T (2021). Effectiveness of mRNA Covid-19 vaccine among U.S. health care personnel. N. Engl. J. Med..

[22] Sadoff J (2021). Safety and efficacy of single-dose Ad26.COV2.S vaccine against Covid-19. N. Engl. J. Med..

[23] Dunkle LM (2022). Efficacy and safety of NVX-CoV2373 in adults in the United States and Mexico. N. Engl. J. Med..

[24] Falsey AR (2021). Phase 3 safety and efficacy of AZD1222 (ChAdOx1 nCoV-19) Covid-19 vaccine. N. Engl. J. Med..

[25] Baden LR (2021). Efficacy and safety of the mRNA-1273 SARS-CoV-2 vaccine. N. Engl. J. Med..

[26] El Sahly HM (2021). Efficacy of the mRNA-1273 SARS-CoV-2 vaccine at completion of blinded phase. N. Engl. J. Med..

[27] Pilishvili T (2021). Interim estimates of vaccine effectiveness of Pfizer-BioNTech and Moderna COVID-19 vaccines among health care personnel—33 U.S. sites, January–March 2021. MMWR Morb. Mortal. Wkly. Rep..

[28] Cavanaugh AM (2021). COVID-19 outbreak associated with a SARS-CoV-2 R.1 lineage variant in a skilled nursing facility after vaccination program—Kentucky, March 2021. MMWR Morb. Mortal. Wkly. Rep..

[29] Kim, S. S. et al. mRNA vaccine effectiveness against COVID-19 among symptomatic outpatients aged ≥16 years in the United States, February–May 2021. *J. Infect. Dis*. jiab451 (2021). 10.1093/infdis/jiab451 PMC8522410 34498052

[30] Lin DY (2022). Effectiveness of Covid-19 vaccines over a 9-month period in North Carolina. N. Engl. J. Med..

[31] Bruxvoort KJ (2022). Real-world effectiveness of the mRNA-1273 vaccine against COVID-19: interim results from a prospective observational cohort study. Lancet Reg. Health Am..

[32] Thomas SJ (2021). Safety and efficacy of the BNT162b2 mRNA Covid-19 vaccine through 6 months. N. Engl. J. Med..

[33] Ju B (2020). Human neutralizing antibodies elicited by SARS-CoV-2 infection. Nature.

[34] Piccoli L (2020). Mapping neutralizing and immunodominant sites on the SARS-CoV-2 spike receptor-binding domain by structure-guided high-resolution serology. Cell.

[35] McCallum M (2021). N-terminal domain antigenic mapping reveals a site of vulnerability for SARS-CoV-2. Cell.

[36] McCallum M (2021). SARS-CoV-2 immune evasion by the B.1.427/B.1.429 variant of concern. Science.

[37] Khoury DS (2021). Neutralizing antibody levels are highly predictive of immune protection from symptomatic SARS-CoV-2 infection. Nat. Med..

[38] Bergwerk M (2021). Covid-19 breakthrough infections in vaccinated health care workers. N. Engl. J. Med..

[39] Feng S (2021). Correlates of protection against symptomatic and asymptomatic SARS-CoV-2 infection. Nat. Med..

[40] Abu-Raddad LJ, Chemaitelly H, Butt AA (2021). Effectiveness of the BNT162b2 Covid-19 vaccine against the B.1.1.7 and B.1.351 variants. N. Engl. J. Med..

[41] Zhou D (2021). Evidence of escape of SARS-CoV-2 variant B.1.351 from natural and vaccine-induced sera. Cell.

[42] Liu Y (2021). Neutralizing activity of BNT162b2-elicited serum. N. Engl. J. Med..

[43] Cele S (2022). Omicron extensively but incompletely escapes Pfizer BNT162b2 neutralization. Nature.

[44] Muik A (2022). Neutralization of SARS-CoV-2 Omicron by BNT162b2 mRNA vaccine-elicited human sera. Science.

[45] Nemet I (2022). Third BNT162b2 vaccination neutralization of SARS-CoV-2 Omicron infection. N. Engl. J. Med..

[46] Ritchie, H. et al. Coronavirus Pandemic (COVID-19). https://ourworldindata.org/coronavirus

[47] Muik A (2022). Neutralization of SARS-CoV-2 Omicron by BNT162b2 mRNA vaccine—elicited human sera. Science.

[48] Pajon R (2022). SARS-CoV-2 Omicron variant neutralization after mRNA-1273 booster vaccination. N. Engl. J. Med..

[49] Chemaitelly, H. et al. Duration of protection of BNT162b2 and mRNA-1273 COVID-19 vaccines against symptomatic SARS-CoV-2 Omicron infection in Qatar. Preprint at https://www.medrxiv.org/content/10.1101/2022.02.07.22270568v1 (2022).

[50] Regev-Yochay, G. et al. Efficacy of a fourth dose of Covid-19 mRNA vaccine against Omicron. *N. Engl. J. Med*. **386**, 1377–1380 (2022). 10.1056/NEJMc2202542 PMC9006792 35297591

[51] El Sahly HM (2021). Efficacy of the mRNA-1273 SARS-CoV-2 vaccine at completion of blinded phase. N. Engl. J. Med..

[52] Feikin DR (2022). Duration of effectiveness of vaccines against SARS-CoV-2 infection and COVID-19 disease: results of a systematic review and meta-regression. Lancet.

[53] Tang P (2021). BNT162b2 and mRNA-1273 COVID-19 vaccine effectiveness against the SARS-CoV-2 Delta variant in Qatar. Nat. Med..

[54] Abu-Raddad LJ, Chemaitelly H, Butt AA, National Study Group for C-V (2021). Effectiveness of the BNT162b2 Covid-19 vaccine against the B.1.1.7 and B.1.351 variants. N. Engl. J. Med..

[55] Chung H (2021). Effectiveness of BNT162b2 and mRNA-1273 covid-19 vaccines against symptomatic SARS-CoV-2 infection and severe covid-19 outcomes in Ontario, Canada: test negative design study. BMJ.

[56] Nasreen, S. et al. Effectiveness of COVID-19 vaccines against symptomatic SARS-CoV-2 infection and severe outcomes with variants of concern in Ontario. *Nat. Microbiol*. **7**, 379–385 (2022). 10.1038/s41564-021-01053-0 35132198

[57] Andrews N (2022). Duration of protection against mild and severe disease by Covid-19 vaccines. N. Engl. J. Med..

[58] Collie S, Champion J, Moultrie H, Bekker LG, Gray G (2022). Effectiveness of BNT162b2 vaccine against Omicron variant in South Africa. N. Engl. J. Med..

[59] Gilbert P, Self S, Rao M, Naficy A, Clemens J (2001). Sieve analysis: methods for assessing from vaccine trial data how vaccine efficacy varies with genotypic and phenotypic pathogen variation. J. Clin. Epidemiol..

[60] Rolland M (2011). Genetic impact of vaccination on breakthrough HIV-1 sequences from the STEP trial. Nat. Med..

[61] Rolland M, Gilbert PB (2021). Sieve analysis to understand how SARS-CoV-2 diversity can impact vaccine protection. PLoS Pathog..

[62] Gupta V, Earl DJ, Deem MW (2006). Quantifying influenza vaccine efficacy and antigenic distance. Vaccine.

[63] Munoz ET, Deem MW (2005). Epitope analysis for influenza vaccine design. Vaccine.

[64] Abu-Raddad LJ (2021). Association of prior SARS-CoV-2 infection with risk of breakthrough infection following mRNA vaccination in Qatar. JAMA.

[65] Andeweg, S. P. et al. Protection of COVID-19 vaccination and previous infection against Omicron BA.1, BA.2 and Delta SARS-CoV-2 infections. Preprint at https://www.medrxiv.org/content/10.1101/2022.02.06.22270457v1 (2022). 10.1038/s41467-022-31838-8 PMC9373894 35961956

[66] Altarawneh, H. N. et al. Effect of prior infection, vaccination, and hybrid immunity against symptomatic BA.1 and BA.2 Omicron infections and severe COVID-19 in Qatar. Preprint at https://www.medrxiv.org/content/10.1101/2022.03.22.22272745v1 (2022).

[67] Shu, Y. & McCauley, J. GISAID: global initiative on sharing all influenza data—from vision to reality. *Euro. Surveill*. **22**, 30494 (2017). 10.2807/1560-7917.ES.2017.22.13.30494 PMC5388101 28382917

[68] Kuznetsova A, Brockhoff PB, Christensen RH (2017). lmerTest package: tests in linear mixed effects models. J. Stat. Softw..

[69] Knowles, J. E., Frederick, C. & Knowles, M. J. E. merTools: Tools for Analyzing Mixed Effect Regression Models. https://cran.r-project.org/web/packages/merTools/index.html (2020).

[70] Lin LI (1989). A concordance correlation coefficient to evaluate reproducibility. Biometrics.

