# Supplementary material for: Rapid evaluation of COVID-19 vaccine effectiveness against symptomatic infection with SARS-CoV-2 variants by analysis of genetic distance
Source: Nat Med. 2022 Jun 16;28(8):1715–22. doi: 10.1038/s41591-022-01877-1 (PMC9388371; doi:10.1038/s41591-022-01877-1)
Supplement: Supplementary file 1 — Supplementary Tables 1–7 and Supplementary Figs. 1 and 2 [file 41591_2022_1877_MOESM1_ESM.pdf]

nature portfolio

https://doi.org/10.1038/s41591-022-01877-1

Supplementary information

Rapid evaluation of COVID-19 vaccine effectiveness against symptomatic infection with SARS-CoV-2 variants by analysis of genetic distance

In the format provided by the authors and unedited

Supplementary Information

Rapid evaluation of COVID-19 vaccine effectiveness against symptomatic infection with SARS-CoV-2 variants by analysis of genetic distance

Table 1: The source of COVID-19 vaccine efficacy or effectiveness (VE) data by December 24, 2021

No.	Developers	Vaccine platforms	Vaccine products	Countries	Epidemiological studies	VE source	Data derived from
1	Bharat Biotech	Inactivated virus	COVAXIN	India	Phase 3 clinical trial	[1]	Table 2
2	Sinopharm + China National Biotec Group Co + Beijing Institute of Biological Products	Inactivated virus	Inactivated SARS-CoV-2 vaccine (Vero cell), vaccine name BBIBP-CorV	United Arab Emirates, Bahrain	Phase 3 clinical trial	[2]	Table 2
3	Sinopharm + China National Biotec Group Co + Wuhan Institute of Biological Products	Inactivated virus	Inactivated SARS-CoV-2 vaccine (Vero cell)	United Arab Emirates, Bahrain	Phase 3 clinical trial	[2]	Table 2
4	Sinovac Research and Development Co. Ltd	Inactivated virus	SARS-CoV-2 vaccine (Inactivated)	Brazil	Phase 3 clinical trial	[3]	Table 2
5				Indonesia	Phase 3 clinical trial	[4]	Table 3
6	BioNTech / Pfizer + Fosun Pharma	RNA based vaccine	BNT162b2 (3 LNP-mRNAs)	Argentina	Phase 3 clinical trial	[5]	Table 3
7				Brazil	Phase 3 clinical trial	[5]	Table 3
8				United States of America	Phase 3 clinical trial	[5]	Table 3
9				Argentina	Phase 3 clinical trial	[6]	Table 3
10				Brazil	Phase 3 clinical trial	[6]	Table 3
11				United States of America	Phase 3 clinical trial	[6]	Table 3
12	Moderna + National Institute of Allergy and Infectious Diseases (NIAID)	RNA based vaccine	mRNA-1273	United States of America	Phase 3 clinical trial	[7]	Figure 4
13				United States of America	Phase 3 clinical trial	[8]	Figure 3
14	Novavax	Protein Subunit	SARS-CoV-2 rS/Matrix M1-Adjuvant (Full length recombinant SARS-CoV-2 glycoprotein nanoparticle vaccine adjuvanted with Matrix M)	Mexico	Phase 3 clinical trial	[9]	Figure 3
15				United States of America	Phase 3 clinical trial	[9]	Figure 3
16				South Africa (B.1.351)	Phase 3 clinical trial	[10]	Text
17				United Kingdom (B.1.1.7)	Phase 3 clinical trial	[11]	Figure 4
18				United Kingdom (Non-B.1.1.7)	Phase 3 clinical trial	[11]	Figure 4
19	CanSino Biological Inc./Beijing Institute of Biotechnology	Viral vector (Non-replicating)	Recombinant novel coronavirus vaccine (Adenovirus type 5 vector)	Argentina	Phase 3 clinical trial	[12]	Table 2
20	Gamaleya Research Institute; Health Ministry of the Russian Federation	Viral vector (Non-replicating)	Gam-COVID-Vac Adeno-based (rAd26-S+rAd5-S)	Russia	Phase 3 clinical trial	[13]	Table 2
21	Janssen Pharmaceutical	Viral vector (Ad26)	Ad26.COV2.S	Argentina, Chile, Colombia, Mexico, Peru	Phase 3 clinical trial	[14]	Table 3
22				Brazil	Phase 3 clinical trial	[14]	Table 3
23				South Africa (B.1.351)	Phase 3 clinical trial	[14]	Table 3
24				United States of America	Phase 3 clinical trial	[14]	Table 3
25		Viral vector (Non-replicating)	AZD1222 (ChAdOx1-S)	Brazil (B.1.1.28)	Phase 3 clinical trial	[15]	Table 2

26	AstraZeneca + University of Oxford			Brazil (P.1)	Phase 3 clinical trial	[15]	Table 2
27				Brazil (P.2)	Phase 3 clinical trial	[15]	Table 2
28				United States of America	Phase 3 clinical trial	[16]	Figure 3
29				Chile, Peru	Phase 3 clinical trial	[16]	Figure 3
30				Brazil	Phase 3 clinical trial	[17]	Table 2
31				United Kingdom	Phase 3 clinical trial	[17]	Table 2
32				United Kingdom, Brazil, South Africa	Phase 3 clinical trial	[18]	Table 1
33				United Kingdom (B.1.1.7)	Phase 3 clinical trial	[19]	Table 1
34	Bharat Biotech	Inactivated virus	COVAXIN	India	Case-control	[20]	Table 2
35				India (Vellore)	Cohort	[21]	Text
36	Sinopharm + China National Biotec Group Co + Beijing Institute of Biological Products<br>Sinovac Research and Development Co. Ltd	Inactivated virus	Inactivated SARS-CoV-2 vaccine (Vero cell), vaccine name BBIBP-CorV<br>SARS-CoV-2 vaccine (Inactivated)	China (Guangzhou) (B.1.617.2)	Cohort	[22]	Table 2
37	Sinovac Research and Development Co. Ltd	Inactivated virus	SARS-CoV-2 vaccine (Inactivated)	Brazil (Manaus)	Case-control	[23]	Text
38				Brazil (São Paulo State)	Case-control	[24]	Table 2
39	BioNTech / Pfizer + Fosun Pharma	RNA based vaccine	BNT162b2 (3 LNP-mRNAs)	Italy	Cohort	[25]	Table 3
40				Israel	Cohort	[26]	Table 2
41				US (Kentucky)	Cohort	[27]	Table 1
42				Italy	Cohort	[28]	Table 2
43				Israel	Cohort	[29]	Table 2b
44				Israel	Cohort	[30]	Table
45				United Kingdom (B.1.1.7)	Case-control	[31]	Table 1
46				United Kingdom (B.1.617.2)	Case-control	[31]	Table 1
47				United Kingdom (B.1.1.7)	Case-control	[32]	Table 2
48				United Kingdom (B.1.617.2)	Case-control	[32]	Table 2
49				United States of America (North Carolina)	Cohort	[33]	Text
50				United States of America	Case-control	[34]	Table 3
51				Italy (Treviso)	Cohort	[35]	Table 2
52				United Kingdom	Case-control	[36]	Text
53				Israel	Cohort	[37]	Table 2
54				United Kingdom (Scotland) (B.1.1.7)	Test-negative case-control	[38]	Table S5

55				United Kingdom (Scotland) (B.1.617.2)	Test-negative case-control	[38]	Table S5
56				Canada	Case-control	[39]	Table 2
57				Israel	Cohort	[40]	Table 4
58				Israel	Cohort	[41]	Text
59	Moderna + National Institute of Allergy and Infectious Diseases (NIAID)	RNA based vaccine	mRNA-1273	United States of America	Case-control	[34]	Table 3
60				United States of America (North Carolina)	Cohort	[33]	Text
61				United States of America (Southern California)	Cohort	[42]	Table 3
62	BioNTech / Pfizer + Fosun Pharma	RNA based vaccine	BNT162b2 (3 LNP-mRNAs)	Canada (Ontario) (Earlier variant)	Test-negative case-control	[43]	eTable 6
63	Moderna + National Institute of Allergy and Infectious Diseases (NIAID)		mRNA-1273	Canada (Ontario) (B.1.1.7)	Test-negative case-control	[43]	eTable 6
64				Canada (Ontario) (B.1.351/P.1)	Test-negative case-control	[43]	eTable 6
65				France (Earlier variant)	Case-control	[44]	Text
66				France (B.1.1.7)	Case-control	[44]	Text
67				France (B.1.351/P.1)	Case-control	[44]	Text
68				United States of America (33 U.S. sites)	Test-negative case-control	[45]	Table 2
69				United States of America (Michigan, Pennsylvania, Texas, Washington, and Wisconsin)	Test-negative case-control	[46]	Table 2
70	Janssen Pharmaceutical	Viral vector (Ad26)	Ad26.COV2.S	United States of America (North Carolina)	Cohort	[33]	Text
71				Brazil (Mato-Grosso do Sul)	Test-negative case-control	[47]	Table 2
72	AstraZeneca + University of Oxford	Viral vector (Non-replicating)	AZD1222 (ChAdOx1-S)	United Kingdom (B.1.617.2)	Case-control	[31]	Table 1
73				India	Case-control	[48]	Table 2
74				United Kingdom (B.1.1.7)	Case-control	[32]	Table 2
75				United Kingdom (B.1.617.2)	Case-control	[32]	Table 2
76				India (Puducherry)	Test-negative case-control	[49]	Table 2
77				United Kingdom (Scotland) (B.1.1.7)	Test-negative case-control	[38]	Table S5
78				United Kingdom (Scotland) (B.1.617.2)	Test-negative case-control	[38]	Table S5

Table 2: COVID-19 VE prediction models for different vaccine platforms based on the genetic distance on the RBD

**Vaccine platforms**		**Slope (95% CI)**	
mRNA		-5.2 (-8.0, -2.4)	
Viral-vector		-6.8 (-9.4, -4.2)	
Protein subunit		-14.3 (-19.2, -9.4)	
Inactivated		-15.8 (-19.3, -12.4)	
**Random effects variance**	146.0	**Two-sided *p*-value for RBD mismatch**	0.038
**Error variance**	38.8	R^2	86.3%

Table 3: COVID-19 VE prediction models for six vaccine products based on the genetic distance on the RBD

**Vaccine products**		**Slope (95% CI)**	
Moderna: mRNA-1273		-4.8 (-7.8, -1.9)	
Pfizer–BioNTech: BNT162b2		-7.5 (-10.9, -4.1)	
Oxford–AstraZeneca: AZD1222		-5.0 (-7.8, -2.2)	
Janssen: Ad26.COV2.S		-8.5 (-12.1, -4.9)	
Sinovac: CoronaVac		-13.6 (-17.1, -10.1)	
Novavax: NVX-CoV2373		-14.1 (-18.2, -10.0)	
**Random effects variance**	145.0	**Two-sided *p*-value for RBD mismatch**	0.006
**Error variance**	26.9	R^2	87.9%

Table 4: COVID-19 VE prediction models for different vaccine platforms based on the genetic distance on the NTD and S protein

**By genetic distance on the NTD:**			
**Vaccine platforms**		**Slope (95% CI)**	
mRNA		-2.5 (-4.6, -0.5)	
Viral-vector		-4.0 (-6.1, -1.8)	
Protein subunit		-5.6 (-9.5, -1.7)	
Inactivated		-7.8 (-10.2, -5.5)	
**Random effects variance**	105.9	**Two-sided *p*-value for NTD mismatch**	0.086
**Error variance**	62.4	R^2	75.8%

**By genetic distance on the S protein:**			
**Vaccine platforms**		**Slope (95% CI)**	
mRNA		-0.8 (-1.7, 0.2)	
Viral-vector		-1.6 (-2.6, -0.6)	
Protein subunit		-2.2 (-4.0, -0.5)	
Inactivated		-4.1 (-5.2, -2.9)	
**Random effects variance**	79.6	**Two-sided *p*-value for S protein mismatch**	0.082
**Error variance**	64.3	R^2	78.4%

Table 5: The source of COVID-19 vaccine efficacy or effectiveness (VE) for validation

No.	Vaccine products	Vaccine Platforms	Observed VE	(95%CI)	References
Alpha (B.1.1.7)					
[1]	BNT162b2	mRNA	97	(96, 98)	31
[2]	BNT162b2	mRNA	93.7	(91.6, 95.3)	32
[3]	BNT162b2	mRNA	92	(88, 94)	38
[4]	BNT162b2	mRNA	90	(85, 94)	43
[5]	BNT162b2	mRNA	86	(81, 90)	44
[6]	AZD1222	Viral vector	81	(72, 87)	38
[7]	AZD1222	Viral vector	74.5	(68.4, 79.4)	32
[8]	AZD1222	Viral vector	70.4	(43.6, 84.5)	19
Beta (B.1.351)					
[9]	BNT162b2	mRNA	77	(63, 86)	44
[10]	Ad26.COV2.S	Viral vector	52	(30.3, 67.4)	14
Gamma (P.1)					
[11]	BNT162b2	mRNA	88	(61, 96)	43
[12]	AZD1222	Viral vector	63.6	(-2.1, 87)	15
Delta (B.1.617.2)					
[13]	BNT162b2	mRNA	88	(85.3, 90.1)	32
[14]	BNT162b2	mRNA	84	(82, 86)	31
[15]	BNT162b2	mRNA	83	(78, 87)	38
[16]	AZD1222	Viral vector	71	(66, 74)	31
[17]	AZD1222	Viral vector	67	(61.3, 71.8)	32
[18]	AZD1222	Viral vector	61	(51, 70)	38
[19]	Covaxin	Inactivated	65.2	(33.1, 83)	1
[20]	CoronaVac	Inactivated	60.4	(31.8, 88.9)	22
B.1.1.28					
[21]	AZD1222	Viral vector	72.6	(46.4, 86)	15
Zeta (P.2)					
[22]	AZD1222	Viral vector	68.7	(54.9, 78.3)	15
Omicron (B.1.1.529)					
[23]	mRNA-1273	mRNA	13.9	(10.5, 17.1)	50

Table 6: SARS-CoV-2 variants tracked by WHO, including variants of concern (VOCs), variants of interest (VOIs), variants under monitoring (VUMs) and formerly monitored variants (March 16, 2022)

Pango lineages	WHO label	Nextstrain clade	GISAID clade	VOI or VOC	Earliest documented samples	Mutations in S protein
B.1.1.7	Alpha	20I (V1)	GRY	VOC	United Kingdom	69-70del, 144del, N501Y, A570D, D614G, P681H, T716I, S982A, D1118H
B.1.351	Beta	20H (V2)	GH/501Y.V2	VOC	South Africa	D80A, D215G, Δ242-244, K417N, E484K, N501Y, D614G, A701V
P.1	Gamma	20J (V3)	GR/501Y.V3	VOC	Brazil	L18F, T20N, P26S, D138Y, R190S, K417T, E484K, N501Y, D614G, H655Y, T1027I, V1176F
B.1.617.2	Delta	21A, 21I, 21J	G/478K.V1	VOC	India	T19R, 157del, L452R, T478K, D614G, P681R, D950N
B.1.1.529	Omicron	21K, 21L, 21M	GRA	VOC	Multiple countries	T19I, L24del, P25del, P26del, A27S, A67V, H69del, V70del, T95I, G142D, V143del, Y144del, Y145del , N211I, L212V, V213R, V213G, ins214EP, R214del, R216E, G339D, R346K, S371F, S371L, S373P, S375F, T376A, D405N, R408S, K417N, N440K, G446S, S477N, T478K, E484A, Q493R, G496S, Q498R, N501Y, Y505H, T547K, D614G, H655Y, N679K, P681H, N764K, D796Y, Q954H, N969K, N856K and L981F,
C.37	Lambda	21G	GR/452Q.V1	VOI	Peru	G75V, T76I, 249del, 251del, 252del, L452Q, F490S, D614G, T859N
B.1.621	Mu	21H	GH	VOI	Colombia	T95I, Y144S, Y145N, R346K, E484K, N501Y, D614G, P681H, D950N
B.1.1.318	-	-	GR	VUM	Multiple countries	T95I, E484K, D614G,P681H, D796H
C.1.2	-	-	GR	VUM	South Africa	P9L,P25L, C136F, R190S, D215G, Y449H, T478K, E484K, N501Y, D614G, H655Y, N679K, T716I
B.1.640	-	-	GH/490R	VUM	Multiple countries	P9L, E96Q, R190S, I210T, D215H, R346S, N394S, Y449N, E484K, F490R, N501Y, D614G, P681H, T859N, D936H, D1139H
AV.1	-	-	GR	Formerly monitored variants	United Kingdom	D80G, T95I, G142D, N439K, E484K, D614G, P681H, I1130V, D1139H
AT.1	-	-	GR	Formerly monitored variants	Russian Federation	P9L, H245P, E484K, D614G, E780K
P.2	Zeta	20B/S.484K	GR/484K.V2	Formerly monitored variants	Brazil	E484K, D614G, V1176F
P.3	Theta	21E	GR/1092K.V1	Formerly monitored variants	Philippines	141-143del, E484K, N501Y, D614G, P681H, E1092K, H1101Y, V1176F
R.1	-	20B	GR	Formerly monitored variants	Multiple countries	W152L, E484K, D614G, G769V
B.1.466.2	-	-	GH	Formerly monitored variants	Indonesia	N439K, D614G, P681R
B.1.1.519	-	20B/S.732A	GR	Formerly monitored variants	Multiple countries	T478K, D614G, P681H, T732A
C.36.3	-	-	GR	Formerly monitored variants	Multiple countries	S12F, W152R, R346S, L452R, D614G, Q677H, A899S
B.1.214.2	-	-	G	Formerly monitored variants	Multiple countries	Q414K, N450K, D614G, T716I
B.1.427/B.1.429	Epsilon	21C	GH/452R.V1	Formerly monitored variants	California, United States of America	S13I, W152C, L452R, D614G

B.1.1.523	-	-	GR	Formerly monitored variants	Multiple countries	F306L, E484K, S494P, D614G, E780A, D839V, T1027I
B.1.619	-	20A/S.126A	G	Formerly monitored variants		I210T, N440K, E484K, D614G, D936N, S939F, T1027I
B.1.620	-	-	G	Formerly monitored variants	Multiple countries	P26S, V126A, H245Y, S477N, E484K, D614G, P681H, T1027I, D1118H
B.1.526	Iota	21F	GH/253G.V1	Formerly monitored variants	New York, United States of America	L5F, T95I, D253G, D614G, A701V
B.1.525	Eta	21D	G/484K.V3	Formerly monitored variants	Multiple countries	Q52R, A67V, 69del, 70del, 144del, E484K, D614G, Q677H, F888L
B.1.617.1	Kappa	21B	G/452R.V3	Formerly monitored variants	India	T95I, G142D, E154K, L452R, E484Q, D614G, P681R, Q1071H
B.1.630	-	-	GH	Formerly monitored variants	Dominican Republic	P9L, C136F, A222V, A243del, L244del, L452R, T478R, E484Q, H655Y, D614G, D950N

Table 7: Randomly selected strains from the variants tracked by WHO to investigate the best candidate vaccine antigens

Pango lineages	Accession number	Strain names	Source
B.1.1.7	EPI_ISL_1718637	hCoV-19/England/ALDP-15182AD/2021	GISAID
B.1.351	EPI_ISL_1534311	hCoV-19/South Africa/NHLS-UCT-GS-B188/2021	GISAID
P.1	EPI_ISL_1495024	hCoV-19/Brazil/MG-LBI246/2021	GISAID
B.1.617.2	EPI_ISL_1704630	hCoV-19/India/MH-ICMR-NIV-INSACOG-GSEQ-1304/2021	GISAID
BA.1	EPI_ISL_9215151	hCoV-19/USA/CA-Curative-120628/2021	GISAID
BA.1.1	EPI_ISL_7877115	hCoV-19/India/un-LNHD6/2021	GISAID
BA.2	EPI_ISL_7971635	hCoV-19/South Africa/NICD-N23195/2021	GISAID
BA.3	EPI_ISL_7747499	hCoV-19/South Africa/CERI-KRISP-K033318/2021	GISAID
C.37	EPI_ISL_1629764	hCoV-19/Peru/LIM-UPCH-0372/2021	GISAID
B.1.621	EPI_ISL_2828025	hCoV-19/Colombia/BOL-INS-VG-2019/2021	GISAID
B.1.1.318	EPI_ISL_7335871	hCoV-19/USA/CA-CDPH-3000035642/2021	GISAID
C.1.2	EPI_ISL_7456442	hCoV-19/South Africa/NICD-N21411/2021	GISAID
B.1.640.1	EPI_ISL_7267558	hCoV-19/France/CVL-CERBAHC-11681217/2021	GISAID
B.1.640.2	EPI_ISL_7412265	hCoV-19/USA/TX-CDC-ASC210487089/2021	GISAID
AT.1	EPI_ISL_3454818	hCoV-19/Russia/OMS-RII-MH22451S/2021	GISAID
AV.1	EPI_ISL_3291235	hCoV-19/England/PHEC-Q303Q1F8/2021	GISAID
P.2	EPI_ISL_1240642	hCoV-19/Brazil/MG-FUNED-49391-21/2021	GISAID
P.3	EPI_ISL_1122458	hCoV-19/Philippines/PH-PGC-02772/2021	GISAID
R.1	EPI_ISL_1793651	hCoV-19/Japan/YCH0165/2021	GISAID
B.1.466.2	EPI_ISL_7472531	hCoV-19/Indonesia/JB-GS-WJHL-ITB-GS-184/2021	GISAID
B.1.1.519	EPI_ISL_7703862	hCoV-19/USA/CA-CDPH-3000109212/2021	GISAID
C.36.3	EPI_ISL_8215718	hCoV-19/Egypt/NRC-627/2021	GISAID
B.1.214.2	EPI_ISL_4370585	hCoV-19/USA/CO-CDC-FG-014845/2021	GISAID
B.1.429	EPI_ISL_1525760	hCoV-19/USA/CA-CDC-FG-015454/2021	GISAID
B.1.1.523	EPI_ISL_8189540	hCoV-19/Russia/MAG-CRIE-L188T0024u/2021	GISAID
B.1.619	EPI_ISL_7667718	hCoV-19/Canada/QC-1nQUP-9969036211/2021	GISAID
B.1.620	EPI_ISL_7120450	hCoV-19/Congo/RC-116/2021	GISAID
B.1.525	EPI_ISL_1729603	hCoV-19/Germany/BY-RKI-I-095380/2021	GISAID
B.1.526	EPI_ISL_1200537	hCoV-19/USA/NY-NYCPHL-003648/2021	GISAID
B.1.617.1	EPI_ISL_1818634	hCoV-19/India/KA-NIMH-SEQ-374/2021	GISAID
B.1.630	EPI_ISL_2828025	hCoV-19/Colombia/BOL-INS-VG-2019/2021	GISAID
B.1	EPI_ISL_1473493	hCoV-19/England/CAMC-145D216/2021	GISAID
B.1.2	EPI_ISL_1553218	hCoV-19/USA/IL-S21WGS344/2021	GISAID
B.1.617.3	EPI_ISL_1704623	hCoV-19/India/MH-ICMR-NIV-INSACOG-GSEQ-1294/2021	GISAID

Fig. 1: Schematic representation of the SARS-CoV-2 genome

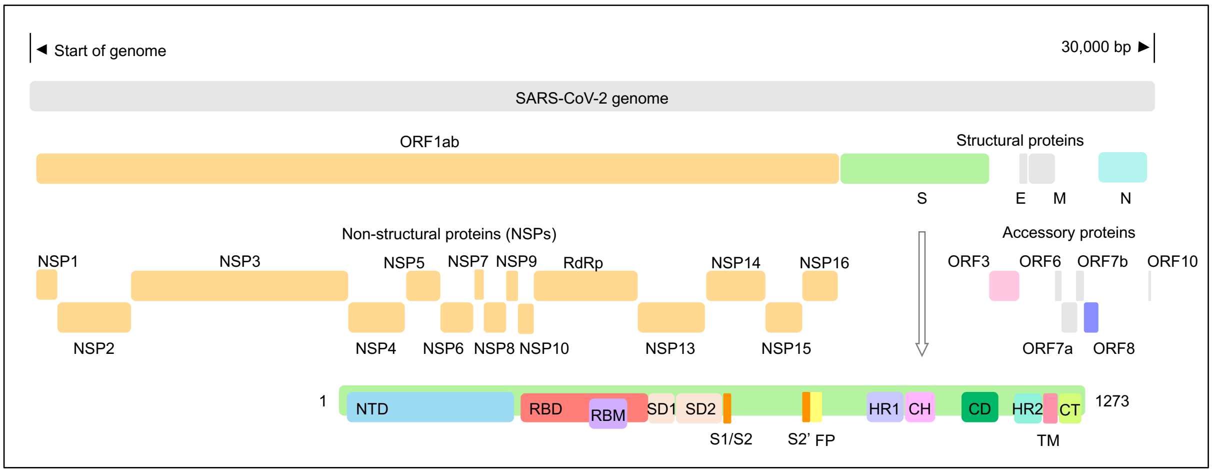

Fig. 2: Structure of the SARS-CoV-2 Spike protein

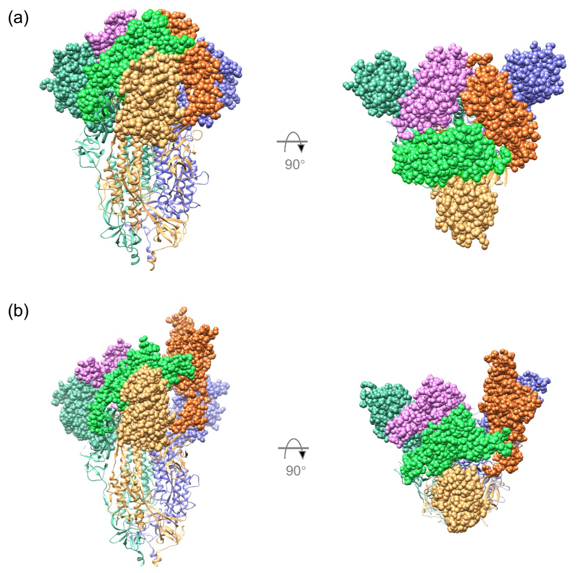

Legend: The location of receptor-binding domain (RBD) and N-terminal domain (NTD) are displayed on the 3D structure of SARS-CoV-2 Spike protein. Panel (a): the conformation of the prefusion trimer; all RBDs in the closed position. Panel (b): the active conformation; one RBD in the open position. The structures are shown from the side view and the top view, respectively. The RBD codons are highlighted in orchid, green and orange spheres. The NTD codons are marked in purple, dark green and yellow. Chimera[51] was used to generate the stereo view of S protein structure with 6VXX[52] and 7DWZ[53] of Protein Data Bank.

References

1. Ella, R., *et al.* Efficacy, safety, and lot-to-lot immunogenicity of an inactivated SARS-CoV-2 vaccine (BBV152): interim results of a randomised, double-blind, controlled, phase 3 trial. *Lancet* **398**, 2173-2184 (2021).
2. Al Kaabi, N., *et al.* Effect of 2 Inactivated SARS-CoV-2 Vaccines on Symptomatic COVID-19 Infection in Adults: A Randomized Clinical Trial. *Jama* **326**, 35-45 (2021).
3. Palacios, R., *et al.* Efficacy and safety of a COVID-19 inactivated vaccine in healthcare professionals in Brazil: the PROFISCOV study. (2021).
4. Fadlyana, E., *et al.* A phase III, observer-blind, randomized, placebo-controlled study of the efficacy, safety, and immunogenicity of SARS-CoV-2 inactivated vaccine in healthy adults aged 18-59 years: An interim analysis in Indonesia. *Vaccine* **39**, 6520-6528 (2021).
5. Polack, F.P., *et al.* Safety and Efficacy of the BNT162b2 mRNA Covid-19 Vaccine. *N Engl J Med* **383**, 2603-2615 (2020).
6. Thomas, S.J., *et al.* Safety and Efficacy of the BNT162b2 mRNA Covid-19 Vaccine through 6 Months. *N Engl J Med* **385**, 1761-1773 (2021).
7. Baden, L.R., *et al.* Efficacy and Safety of the mRNA-1273 SARS-CoV-2 Vaccine. *N Engl J Med* **384**, 403-416 (2021).
8. El Sahly, H.M., *et al.* Efficacy of the mRNA-1273 SARS-CoV-2 Vaccine at Completion of Blinded Phase. *N Engl J Med* **385**, 1774-1785 (2021).
9. Dunkle, L.M., *et al.* Efficacy and Safety of NVX-CoV2373 in Adults in the United States and Mexico. *N Engl J Med* **386**, 531-543 (2022).
10. Shinde, V., *et al.* Efficacy of NVX-CoV2373 Covid-19 Vaccine against the B.1.351 Variant. *N Engl J Med* **384**, 1899-1909 (2021).
11. Heath, P.T., *et al.* Efficacy of the NVX-CoV2373 Covid-19 Vaccine Against the B.1.1.7 Variant. *medRxiv*, 2021.05.13.21256639 (2021).
12. Halperin, S.A., *et al.* Final efficacy analysis, interim safety analysis, and immunogenicity of a single dose of recombinant novel coronavirus vaccine (adenovirus type 5 vector) in adults 18 years and older: an international, multicentre, randomised, double-blinded, placebo-controlled phase 3 trial. *Lancet* **399**, 237-248 (2022).
13. Logunov, D.Y., *et al.* Safety and efficacy of an rAd26 and rAd5 vector-based heterologous prime-boost COVID-19 vaccine: an interim analysis of a randomised controlled phase 3 trial in Russia. *Lancet* **397**, 671-681 (2021).
14. Sadoff, J., *et al.* Safety and Efficacy of Single-Dose Ad26.COV2.S Vaccine against Covid-19. *N Engl J Med* **384**, 2187-2201 (2021).
15. Clemens, S.A.C., *et al.* Efficacy of ChAdOx1 nCoV-19 (AZD1222) vaccine against SARS-CoV-2 lineages circulating in Brazil. *Nat Commun* **12**, 5861 (2021).
16. Falsey, A.R., *et al.* Phase 3 Safety and Efficacy of AZD1222 (ChAdOx1 nCoV-19) Covid-19 Vaccine. *N Engl J Med* **385**, 2348-2360 (2021).
17. Voysey, M., *et al.* Safety and efficacy of the ChAdOx1 nCoV-19 vaccine (AZD1222) against SARS-CoV-2: an interim analysis of four randomised controlled trials in Brazil, South Africa, and the UK. *Lancet* **397**, 99-111 (2021).
18. Voysey, M., *et al.* Single-dose administration and the influence of the timing of the booster dose on immunogenicity and efficacy of ChAdOx1 nCoV-19 (AZD1222) vaccine: a pooled analysis of four randomised trials. *Lancet* **397**, 881-891 (2021).

19. Emary, K.R.W., *et al.* Efficacy of ChAdOx1 nCoV-19 (AZD1222) vaccine against SARS-CoV-2 variant of concern 202012/01 (B.1.1.7): an exploratory analysis of a randomised controlled trial. *Lancet* **397**, 1351-1362 (2021).

20. Desai, D., *et al.* Effectiveness of an inactivated virus-based SARS-CoV-2 vaccine, BBV152, in India: a test-negative, case-control study. *Lancet Infect Dis* **22**, 349-356 (2022).

21. Murugesan, M., *et al.* Protective effect conferred by prior infection and vaccination on COVID-19 in a Healthcare Worker Cohort in South India. *Available at SSRN 3914633* (2021).

22. Kang, M., *et al.* Effectiveness of Inactivated COVID-19 Vaccines Against Illness Caused by the B.1.617.2 (Delta) Variant During an Outbreak in Guangdong, China : A Cohort Study. *Ann Intern Med* (2022).

23. Hitchings, M.D.T., *et al.* Effectiveness of CoronaVac among healthcare workers in the setting of high SARS-CoV-2 Gamma variant transmission in Manaus, Brazil: A test-negative case-control study. *Lancet Reg Health Am* **1**, 100025 (2021).

24. Ranzani, O.T., *et al.* Effectiveness of the CoronaVac vaccine in older adults during a gamma variant associated epidemic of covid-19 in Brazil: test negative case-control study. *Bmj* **374**, n2015 (2021).

25. Bianchi, F.P., *et al.* BNT162b2 mRNA COVID-19 Vaccine Effectiveness in the Prevention of SARS-CoV-2 Infection and Symptomatic Disease in Five-Month Follow-Up: A Retrospective Cohort Study. *Vaccines (Basel)* **9**(2021).

26. Dagan, N., *et al.* BNT162b2 mRNA Covid-19 Vaccine in a Nationwide Mass Vaccination Setting. *N Engl J Med* **384**, 1412-1423 (2021).

27. Cavanaugh, A.M., *et al.* COVID-19 Outbreak Associated with a SARS-CoV-2 R.1 Lineage Variant in a Skilled Nursing Facility After Vaccination Program - Kentucky, March 2021. *MMWR Morb Mortal Wkly Rep* **70**, 639-643 (2021).

28. Giansante, C., *et al.* COVID-19 vaccine effectiveness among the staff of the Bologna Health Trust, Italy, December 2020-April 2021. *Acta Biomed* **92**, e2021270 (2021).

29. Regev-Yochay, G., *et al.* Decreased infectivity following BNT162b2 vaccination: A prospective cohort study in Israel. *Lancet Reg Health Eur* **7**, 100150 (2021).

30. Katz, M.A., *et al.* Early effectiveness of BNT162b2 Covid-19 vaccine in preventing SARS-CoV-2 infection in healthcare personnel in six Israeli hospitals (CoVEHPI). *Vaccine* **40**, 512-520 (2022).

31. Pouwels, K.B., *et al.* Effect of Delta variant on viral burden and vaccine effectiveness against new SARS-CoV-2 infections in the UK. *Nat Med* **27**, 2127-2135 (2021).

32. Lopez Bernal, J., *et al.* Effectiveness of Covid-19 Vaccines against the B.1.617.2 (Delta) Variant. *N Engl J Med* **385**, 585-594 (2021).

33. Lin, D., *et al.* Effectiveness of Covid-19 vaccines in the United States over 9 months: surveillance data from the state of North Carolina. *medRxiv* (2021).

34. Pilishvili, T., *et al.* Effectiveness of mRNA Covid-19 Vaccine among U.S. Health Care Personnel. *N Engl J Med* **385**, e90 (2021).

35. Fabiani, M., *et al.* Effectiveness of the Comirnaty (BNT162b2, BioNTech/Pfizer) vaccine in preventing SARS-CoV-2 infection among healthcare workers, Treviso province, Veneto region, Italy, 27 December 2020 to 24 March 2021. *Euro Surveill* **26**(2021).

36. Lopez Bernal, J., *et al.* Effectiveness of the Pfizer-BioNTech and Oxford-AstraZeneca vaccines on covid-19 related symptoms, hospital admissions, and mortality in older adults in England: test negative case-control study. *Bmj* **373**, n1088 (2021).

37. Haas, E.J., *et al.* Impact and effectiveness of mRNA BNT162b2 vaccine against SARS-CoV-2 infections and COVID-19 cases, hospitalisations, and deaths following a nationwide

vaccination campaign in Israel: an observational study using national surveillance data. *Lancet* **397**, 1819-1829 (2021).

38. Sheikh, A., McMenamin, J., Taylor, B. & Robertson, C. SARS-CoV-2 Delta VOC in Scotland: demographics, risk of hospital admission, and vaccine effectiveness. *Lancet* **397**, 2461-2462 (2021).

39. Carazo, S.*, et al.* Single-dose mRNA vaccine effectiveness against SARS-CoV-2 in healthcare workers extending 16 weeks post-vaccination: a test-negative design from Quebec, Canada. *Clin Infect Dis* (2021).

40. Glatman-Freedman, A., Bromberg, M., Dichtiar, R., Hershkovitz, Y. & Keinan-Boker, L. The BNT162b2 vaccine effectiveness against new COVID-19 cases and complications of breakthrough cases: A nation-wide retrospective longitudinal multiple cohort analysis using individualised data. *EBioMedicine* **72**, 103574 (2021).

41. Chodick, G.*, et al.* The Effectiveness of the Two-Dose BNT162b2 Vaccine: Analysis of Real-World Data. *Clin Infect Dis* **74**, 472-478 (2022).

42. Bruxvoort, K.J.*, et al.* Real-world effectiveness of the mRNA-1273 vaccine against COVID-19: Interim results from a prospective observational cohort study. *Lancet Reg Health Am* **6**, 100134 (2022).

43. Chung, H.*, et al.* Effectiveness of BNT162b2 and mRNA-1273 covid-19 vaccines against symptomatic SARS-CoV-2 infection and severe covid-19 outcomes in Ontario, Canada: test negative design study. *Bmj* **374**, n1943 (2021).

44. Charmet, T.*, et al.* Impact of original, B.1.1.7, and B.1.351/P.1 SARS-CoV-2 lineages on vaccine effectiveness of two doses of COVID-19 mRNA vaccines: Results from a nationwide case-control study in France. *Lancet Reg Health Eur* **8**, 100171 (2021).

45. Pilishvili, T.*, et al.* Interim Estimates of Vaccine Effectiveness of Pfizer-BioNTech and Moderna COVID-19 Vaccines Among Health Care Personnel - 33 U.S. Sites, January-March 2021. *MMWR Morb Mortal Wkly Rep* **70**, 753-758 (2021).

46. Kim, S.S.*, et al.* mRNA Vaccine Effectiveness against COVID-19 among Symptomatic Outpatients Aged ≥16 Years in the United States, February - May 2021. *J Infect Dis* (2021).

47. Ranzani, O.T.*, et al.* Vaccine effectiveness of Ad26. COV2. S against symptomatic COVID-19 and clinical outcomes in Brazil: a test-negative study design. *medRxiv* (2021).

48. Thiruvengadam, R.*, et al.* Effectiveness of ChAdOx1 nCoV-19 vaccine against SARS-CoV-2 infection during the delta (B.1.617.2) variant surge in India: a test-negative, case-control study and a mechanistic study of post-vaccination immune responses. *Lancet Infect Dis* (2021).

49. Pramod, S., Govindan, D., Ramasubramani, P., Kar, S.S. & Aggarwal, R. Effectiveness of Covishield vaccine in preventing Covid-19 - A test-negative case-control study. *Vaccine* (2022).

50. Tseng, H.F.*, et al.* Effectiveness of mRNA-1273 against SARS-CoV-2 Omicron and Delta variants. *Nat Med* (2022).

51. Pettersen, E.F.*, et al.* UCSF Chimera--a visualization system for exploratory research and analysis. *J Comput Chem* **25**, 1605-12 (2004).

52. Ke, Z.*, et al.* Structures and distributions of SARS-CoV-2 spike proteins on intact virions. *Nature* **588**, 498-502 (2020).

53. Yan, R.*, et al.* Structural basis for the different states of the spike protein of SARS-CoV-2 in complex with ACE2. *Cell Res* **31**, 717-719 (2021).
