# Supplementary material for: Rapid evaluation of COVID-19 vaccine effectiveness against symptomatic infection with SARS-CoV-2 variants by analysis of genetic distance
Source: Nat Med. 2022 Jun 16;28(8):1715–22. doi: 10.1038/s41591-022-01877-1 (PMC9388371; doi:10.1038/s41591-022-01877-1)
Supplement: Supplementary file 2 — Reporting Summary [file 41591_2022_1877_MOESM2_ESM.pdf]

nature portfolio

Corresponding author(s): Maggie Haitian Wang

Last updated by author(s): May 11, 2022



# Reporting Summary

Nature Portfolio wishes to improve the reproducibility of the work that we publish. This form provides structure for consistency and transparency in reporting. For further information on Nature Portfolio policies, see our Editorial Policies and the Editorial Policy Checklist.

## Statistics

For all statistical analyses, confirm that the following items are present in the figure legend, table legend, main text, or Methods section.

| n/a | Confirmed | |
|---|---|---|
| ☐ | ☒ | The exact sample size (*n*) for each experimental group/condition, given as a discrete number and unit of measurement |
| ☐ | ☒ | A statement on whether measurements were taken from distinct samples or whether the same sample was measured repeatedly |
| ☐ | ☒ | The statistical test(s) used AND whether they are one- or two-sided<br>*Only common tests should be described solely by name; describe more complex techniques in the Methods section.* |
| ☐ | ☒ | A description of all covariates tested |
| ☒ | ☐ | A description of any assumptions or corrections, such as tests of normality and adjustment for multiple comparisons |
| ☐ | ☒ | A full description of the statistical parameters including central tendency (e.g. means) or other basic estimates (e.g. regression coefficient) AND variation (e.g. standard deviation) or associated estimates of uncertainty (e.g. confidence intervals) |
| ☐ | ☒ | For null hypothesis testing, the test statistic (e.g. *F*, *t*, *r*) with confidence intervals, effect sizes, degrees of freedom and *P* value noted<br>*Give P values as exact values whenever suitable.* |
| ☒ | ☐ | For Bayesian analysis, information on the choice of priors and Markov chain Monte Carlo settings |
| ☐ | ☒ | For hierarchical and complex designs, identification of the appropriate level for tests and full reporting of outcomes |
| ☐ | ☒ | Estimates of effect sizes (e.g. Cohen's *d*, Pearson's *r*), indicating how they were calculated |

*Our web collection on statistics for biologists contains articles on many of the points above.*

## Software and code

Policy information about availability of computer code

| | |
|---|---|
| Data collection | No software was used for data collection. |
| Data analysis | We used MAFFT (version 7) for multiple sequence alignment and R statistical software (version 4.0.3) in all statistical analyses. R packages used in this study include lmerTest (3.1-3) and merTools(0.5.2). All code is freely available at https://github.com/VaccineEffectivenessPrediction/COVID19-Vaccine-Effectiveness. |

For manuscripts utilizing custom algorithms or software that are central to the research but not yet described in published literature, software must be made available to editors and reviewers. We strongly encourage code deposition in a community repository (e.g. GitHub). See the Nature Portfolio guidelines for submitting code & software for further information.

## Data

Policy information about availability of data

All manuscripts must include a data availability statement. This statement should provide the following information, where applicable:

- Accession codes, unique identifiers, or web links for publicly available datasets
- A description of any restrictions on data availability
- For clinical datasets or third party data, please ensure that the statement adheres to our policy

All data used in this study is publicly available. The detailed information of VE outcomes is available in the Supplementary Information. Viral sequence data were downloaded from the global initiative on sharing all influenza data (GISAID) at http://platform.gisaid.org/ and the accession numbers are provided in the online Supplementary Acknowledgment Table (https://github.com/VaccineEffectivenessPrediction/COVID19-Vaccine-Effectiveness).

# Field-specific reporting

Please select the one below that is the best fit for your research. If you are not sure, read the appropriate sections before making your selection.

☒ Life sciences ☐ Behavioural & social sciences ☐ Ecological, evolutionary & environmental sciences

For a reference copy of the document with all sections, see nature.com/documents/nr-reporting-summary-flat.pdf

# Life sciences study design

All studies must disclose on these points even when the disclosure is negative.

| | |
|---|---|
| Sample size | The study extracted vaccine efficacy or vaccine effectiveness (VE) data before 24 Dec, 2021 from published articles and preprint articles. A total of 78 VE data were obtained for model building. All available sequences that matched to the period and locations of the clinical trials or observational studies totaled 1,984,241 full-length genome sequences from 31 geographical regions. |
| Data exclusions | For VE data, exclusion criteria include: target population has special conditions; the primary outcome is not symptomatic COVID-19 infection after the second vaccine dose; and the study period of VE evaluation is not reported. For sequence data, strains with duplicated names and unclear collection time of samples were removed. |
| Replication | This study demonstrated a clear relationship between COVID-19 VE and genetic distance on RBD, NTD and entire S protein. Our findings can be supported by biological experiments. We first collected data before June 2021 and determined genetic distance is associated with VE against symptomatic infection. After adding subsequent data before Match 2022, the results are consistent with previous results. Such relationships exist in different vaccine platforms and vaccine products. The prediction results were validated by independent data. All attempts at replication were successful. Additionally, this bioinformatics framework has been applied to influenza A/H1N1pdm09, H3N2 and influenza B viruses and such a relationship was also detected. |
| Randomization | Randomization is not applicable in our study design. The vaccine efficacy outcomes included in this study were based upon clinical trials. The vaccine effectiveness outcomes were obtained from observational studies. All available sequences that matched to the period and locations of the clinical trials or observational studies were collected. |
| Blinding | Blinding is not relevant to the study. This study used population-level data and did not involve individual participants. |

# Reporting for specific materials, systems and methods

We require information from authors about some types of materials, experimental systems and methods used in many studies. Here, indicate whether each material, system or method listed is relevant to your study. If you are not sure if a list item applies to your research, read the appropriate section before selecting a response.

## Materials & experimental systems

| n/a | Involved in the study | |
|---|---|---|
| ☒ | ☐ | Antibodies |
| ☒ | ☐ | Eukaryotic cell lines |
| ☒ | ☐ | Palaeontology and archaeology |
| ☒ | ☐ | Animals and other organisms |
| ☒ | ☐ | Human research participants |
| ☒ | ☐ | Clinical data |
| ☒ | ☐ | Dual use research of concern |

## Methods

| n/a | Involved in the study | |
|---|---|---|
| ☒ | ☐ | ChIP-seq |
| ☒ | ☐ | Flow cytometry |
| ☒ | ☐ | MRI-based neuroimaging |
